# Porcine Rotaviruses: Epidemiology, Immune Responses and Control Strategies

**DOI:** 10.3390/v9030048

**Published:** 2017-03-18

**Authors:** Anastasia N. Vlasova, Joshua O. Amimo, Linda J. Saif

**Affiliations:** 1 Food Animal Health Research Program, CFAES, Ohio Agricultural Research and Development Center, Department of Veterinary Preventive Medicine, The Ohio State University, Wooster, OH 44691, USA; 2 Department of Animal Production, Faculty of Veterinary Medicine, University of Nairobi, Nairobi 30197, Kenya; jamimo@uonbi.ac.ke; 3 Bioscience of Eastern and Central Africa, International Livestock Research Institute (BecA-ILRI) Hub, Nairobi 30709, Kenya

**Keywords:** Porcine rotavirus, group A, B, C, E and H rotaviruses, rotavirus vaccines, epidemiology, genetic variability, prevalence, active and passive immunity, swine, zoonotic potential

## Abstract

Rotaviruses (RVs) are a major cause of acute viral gastroenteritis in young animals and children worldwide. Immunocompetent adults of different species become resistant to clinical disease due to post-infection immunity, immune system maturation and gut physiological changes. Of the 9 RV genogroups (A–I), RV A, B, and C (RVA, RVB, and RVC, respectively) are associated with diarrhea in piglets. Although discovered decades ago, porcine genogroup E RVs (RVE) are uncommon and their pathogenesis is not studied well. The presence of porcine RV H (RVH), a newly defined distinct genogroup, was recently confirmed in diarrheic pigs in Japan, Brazil, and the US. The complex epidemiology, pathogenicity and high genetic diversity of porcine RVAs are widely recognized and well-studied. More recent data show a significant genetic diversity based on the VP7 gene analysis of RVB and C strains in pigs. In this review, we will summarize previous and recent research to provide insights on historic and current prevalence and genetic diversity of porcine RVs in different geographic regions and production systems. We will also provide a brief overview of immune responses to porcine RVs, available control strategies and zoonotic potential of different RV genotypes. An improved understanding of the above parameters may lead to the development of more optimal strategies to manage RV diarrheal disease in swine and humans.

## 1. Introduction

**Figure 1:**
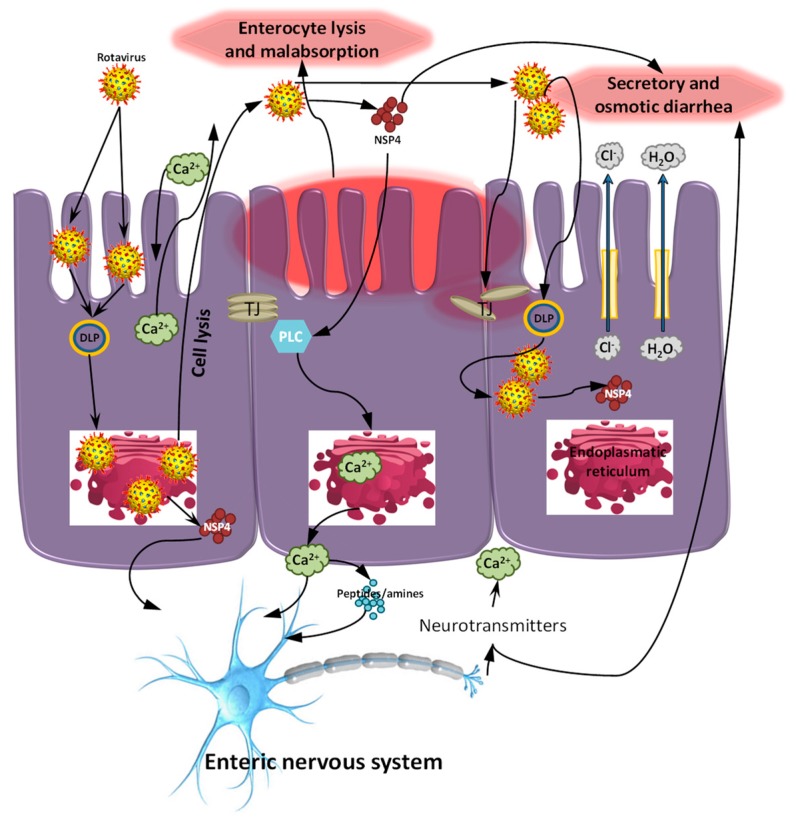
Potential mechanisms of rotavirus (RV) pathogenesis. RV replication inside enterocytes induces osmotic diarrhea. RV also increases the concentration of intracellular calcium (Ca^2+^), disrupting the cytoskeleton and the tight junctions, increasing paracellular permeability. In addition, RV produces non-structural protein 4 (NSP4), an enterotoxin that induces Ca^2+^ efflux from endoplasmatic reticulum via the phospholipase C dependent (PLC) mechanism further contributing to electrolyte imbalance and secretory diarrhea. RV can also stimulate the enteric nervous system (ENS, via NSP4 dependent mechanism), further contributing to secretory diarrhea and increasing intestinal motility. Agents that can inhibit the ENS could be useful in alleviating RV diarrhea in children. Following, tryptic cleavage of viral protein 8 (VP8) from VP5, the VP8 fragment alters the localization of claudin-3, ZO-1 and occludin leading to the disruption of the barrier integrity of tight junctions (TJ) [3,4,5,6]. Late in the infectious process, RV destroys mature enterocytes, further contributing to malabsorptive or osmotic diarrhoea. RV antigens, genomic RNA and infectious particles have been found in the blood of children and blood and systemic organs in animals [7,8]. The role of systemic RV translocation in disease pathogenesis is currently unknown. DLP: double-layered particles.

Rotavirus (RV) is well established as a major cause of acute gastroenteritis in young children and animals, including nursing and weaned piglets [1]. The name “rotavirus” comes from the wheel-like virion appearance observed by electron microscopy. The virus is transmitted by the fecal–oral route and the infection results in destruction of mature small intestinal enterocytes [2]. RV-mediated damage is characterized by shortened villi with sparse, irregular microvilli and by mononuclear cell infiltration of the lamina propria [2]. Several mechanisms are suggested to contribute to the development of diarrhea including malabsorption due to the destruction of enterocytes, villus ischaemia, neuro-regulatory release of a vasoactive agent from infected epithelial cells. Also the RV non-structural protein 4 (NSP4) induces an age- and dose-dependent diarrheal response by acting as an enterotoxin and secretory agonist [2] (Figure 1) to: (i) stimulate Ca^2+^-dependent cell permeability and (ii) alter the integrity of epithelial barrier. 

RVs represent a genus in the *Reoviridae* family of double-stranded RNA (dsRNA) viruses, with a genome of 11 segments of dsRNA encoding six structural viral proteins (VP1–VP4, VP6 and VP7) and five nonstructural proteins (NSP1–NSP5/6). RVs are classified into 10 groups (A–J) based on antigenic relationships of their VP6 proteins, with provisional I and J species recently identified in sheltered dogs in Hungary and in bats in Serbia, respectively [9,10,11,12]. The outer capsid proteins, VP7 and VP4, induce neutralizing antibodies and form the basis for the G and P dual typing system [9]. The most common groups that infect humans and animals are groups A, B and C (RVA, RVB and RVC), with the highest prevalence of RVA strains that represent one of the most significant causes of acute dehydrating diarrhea from public health and veterinary health perspectives. To date, 27 different G- and 37 P-genotypes have been described in both humans and animals for RVAs [13,14]. For highly genetically diverse RVA strains, the dual (G/P) typing system was extended in 2008 to a full-genome sequence classification system, with nucleotide percent identity cut-off values established for all 11 gene segments, with the notations Gx-P[x]-Ix-Rx-Cx-Mx-Ax-Nx-Tx-Ex-Hx used for the VP7-VP4-VP6-VP1-VP2-VP3-NSP1-NSP2-NSP3-NSP4-NSP5/6 encoding genes, respectively [15]. Subsequently, a Rotavirus Classification Working Group (RCWG) was formed to set the RVA classification guidelines and maintain the proposed classification system [16] to facilitate complete classification of novel RVA strains. Currently, only RVA classification has been developed and is being maintained by the RCWG, while much less is known about the epidemiology and disease burden associated with infection by non-RVAs. However, RVB, RVC, RVE, RVH and RVI have been detected in sporadic, endemic or epidemic infections of various mammalian species, whereas RVD, RVF and RVG are found in poultry, such as chickens and turkeys [14,17,18,19,20,21,22,23,24]. RVs of groups A, B, C, E and H have been described in pigs [25,26,27,28,29,30,31,32]. 

In 1969, bovine RV was the first group A RV isolated in cell culture and confirmed as a cause of diarrhea in calves [33,34]. Human RV was discovered soon after, in 1973, by Bishop and colleagues [35]. Subsequent studies documented the widespread prevalence of RVA infections in young animals, including calves and pigs, and their association with diarrhea in animals <1 month of age [20,28,30,36,37]. Group C RVs were first isolated in piglets in 1980 [31] and were subsequently identified in other animals and humans [30,38,39,40,41]. Porcine RVB was first described as an RV-like agent identified in a diarrheic pig in the 1980s [29,42]. In addition to pigs, RVB strains have been also detected in cattle [43,44,45,46], lambs [47], and rats [48]. In contrast to human RVA and RVC that were described worldwide, human RVB strains have been described only in China [49,50,51,52], India [53,54], and Bangladesh [55,56,57,58,59]. An atypical group E porcine RV was only reported in UK swine, where a serological survey indicated a widespread distribution of antibodies to this virus in pigs older than 10 weeks [25,60]. Most recently, RVH strains were described in pigs in Japan, Brazil and in the US, where they were reportedly circulating since at least 2002 [27,61,62]. 

## 2. RV Genogroup/Genotype Classification and Prevalence in Swine

Infections by RVAs are confirmed in pigs worldwide with or without association with diarrhea [63,64,65,66,67,68,69,70,71,72,73,74]. RVA prevalence rates in pigs vary from 3.3% to 67.3% without evidence of seasonality, but with spatio-temporal fluctuations and re-emergence of certain genotypes, including G9 and G1 [67,71,75,76,77,78,79,80,81,82,83,84,85,86,87], with farm-level prevalence reaching 61%–74% [73,74]. Twelve G genotypes (G1 to G6, G8 to G12, and G26) and 16 P genotypes (P[1] to P[8], P[11], P[13], P[19], P[23], P[26], P[27], P[32], and P[34]) of RVA have been associated with pigs [65,67,70,72,73,74,84,88,89,90,91]. However, G3, G4, G5, G9 and G11 were historically considered the most common G genotypes in swine and were usually associated with P[5], P[6], P[7], P[13] and P[28] [16,89,92].

Similar to RVA, porcine RVCs are reported in most parts of the world [32,39]. Diarrhea outbreaks associated with RVCs have been documented in nursing, weaning and post-weaning pigs [31,32,93], either alone or in mixed infection with other enteric pathogens [1]. In addition, the antibody prevalence in pigs (58%–100%) shows that RVC infection may be very common and has circulated for many decades in swine herds in developed countries [32]. Recent studies on US and Canadian porcine samples demonstrated a 46% prevalence of RVC which was higher in very young (78%, ≤3 day old) and young (65%, 4–20 day old) piglets [94]. RVC genotypes G1 and G3 were initially assigned to the prototype porcine RVC Cowden and HF strains, respectively [95]. Further efforts to classify RVC strains into sequence-based genotypes resulted in identification of a total of nine G genotypes (G1–G9), seven P genotypes (P1–P7) and seven I genotypes (I1–I7) [94,96,97,98,99,100]. Additional attempts were made to extend RVC classification based on the sequencing of all 11 genes [101,102]; however, only limited genomic sequences are currently available. Porcine RVCs belong to G1, G3, G5–G9 genotypes and a newly described G10 genotype [103], while bovine and human RVCs are classified as G2 and G4 genotypes, respectively [94,96,97,104]. Additionally, two provisional G genotypes (G12 and G13 based on the 86% nucleotide identity cut-off value) are proposed by Niira et al. based on their recent results [105].

Rapid molecular characterization of RVB strains is hampered by the difficulty of adapting RVB strains to cell culture [32,58]. Additionally, limited and variable fecal shedding and instability in feces were shown for RVBs [44]. Complete genome sequences were obtained for several human RVB strains from Southeast Asia [55,106,107,108] and partial genome sequencing was done for several rat and bovine RVB strains [43,44,45,46,48,53,57,109]. Kuga and colleagues analyzed sequences of the VP7 gene of 38 porcine RVB strains from Japan (2000–2007) and the five genotypes proposed were further divided into 12 clusters, using 67% and 76% nucleotide cut-off values (66% and 79% on the amino acid level, respectively) [110]. Recent results by Marthaler et al. suggested a broader diversity of porcine RVBs based on sequencing of the VP7 gene of 68 RVB strains (collected in 2009 from 14 US states and Japan) defining 20 G genotypes based on an 80% nucleotide identity cut-off value and providing the first evidence that porcine RVB genotypes may be host species- and region-specific [111]. Therefore, porcine RVB strains of genotypes G1, G2 and G3/G5 are only found in rats, humans and bovine species, respectively, while genotypes G4, G7, G9, G13, G15 and G19 are only confirmed in pigs in Japan, and a small number of porcine RVB strains of genotypes G10 and G17 are only found in the US. An additional G genotype, G21, was detected in pigs in India [112]. 

Three human RVH strains from Asia (ADRV-N, J19, B219) [113,114,115,116] and a porcine RVH strain (SKA-1) were identified during 1997–2002 [27]. In 2012, three more porcine RVH strains BR63, BR60, and BR59 from Brazil were identified [62]. Surprisingly high prevalence (15%) of porcine RVH strains was recently demonstrated by Marthaler and colleagues mostly in older (21–55 days old) piglets [18]. Their data suggested that porcine RVH strains circulated in the US herds since at least 2006 and that they are evolutionarily distinct from those of humans, as well as from porcine RVH strains in Brazil and Japan [18]. Complete genome analyses of a porcine RVH identified in South Africa showed that the novel RVH strain MRC-DPRU1575 clustered together with the SKA-1 strain and known porcine RVH strains from Brazil and the USA (only for available genome segments) [117]. However, it was only distantly related to human RVH strains from Asia and an RVH-like strain recently detected in bats from Cameroon [117].

Additional data is needed to evaluate the epidemiological importance of porcine RVE strains, because porcine RVE has only been identified in the UK approximately 3 decades ago and has not been reported to expand its geographic or host range since [25]. 

## 3. Porcine RV Distribution, Genotype Prevalence and Spatio-Temporal Variations in the Americas

### 3.1. North America 

**Figure 2:**
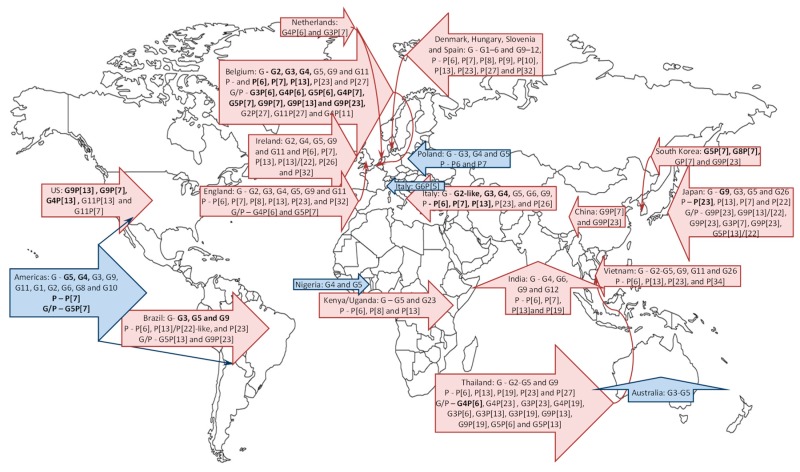
Global genotype distribution of porcine RVA strains reported in historic (1976–2011, blue figure arrows) and current (after 2000, pink figure arrows) studies. Porcine RVAs are also detected in Germany and Russia, but no genotyping data is available.

A high prevalence of porcine RV strains of groups A, B and C among samples from diarrheic piglets collected in 2009–2011 in the US, Canada and Mexico was reported by Marthaler et al. (2014) and Homwong et al. (2016) [69,71]. The highest overall prevalence of porcine RVs of 82.1% (90%–100% for UT, PA, VA and NC, and 5%–90% for the rest of the states) was observed in the US, and similar values of 79.7% and 73.3% are reported for Canada and Mexico, respectively. In the US, the highest proportion of RVA positive samples (70.1%) was in the Southeastern states, whereas the highest detection rate of RVB and RVC samples was found in the South-central states (34.2% and 62.2%, respectively); however no genotyping results were reported. The historic prevalence of porcine RVA, RVB and RVC strains in the US was reported as 67.8%, 10.0% and 11.1%, respectively [26]. A systematic review by Papp and colleagues [72] summarized genotype prevalence and distribution for porcine historic samples collected/analysed between 1976 and 2011 from both diarrheic and non-diarrheic animals. The most prevalent G type of porcine RVA in the Americas was G5 (71.4%), followed by G4 (8.2%), G3 (3.57%), G9 (2.31%) and G11 (1.9%) [68,72,82,118] (Figure 2). The frequencies of infections by other RVA genotypes found in pigs (G1, G2, G6, G8 and G10) were ~1% or less. P[7] genotype was the most common in the Americas (77.2%), while other P-types represented less than 1% of the identified RVA strains [72]. Finally, G5P[7] was the single most prevalent combination. In contrast, the analysis of more recent US RVA strains (2004–2012) conducted by Amimo and colleagues demonstrated that the dominant G-P combination was G9P[13] found in 60.9% of positive samples [from Ohio (OH) North Carolina (NC) and Michigan (MI)], followed by G9P[7] (8.7%), G4P[13] (8.7%), G11P[13] (4.3%), and G11P[7] (4.3%), while no G5 strains were detected [67]. Additionally, despite the relatively low overall prevalence of porcine RVA strains in samples from US diarrheic and non-diarrheic animals of 9.4%, Amimo et al. reported that there was an increase in RVA detection from 5.9% in 2004 to 13.8% in 2012 [67], which may be due to the increase in the prevalence of novel or re-emerging genotypes (such as G9) because of lack of herd immunity against them.

**Figure 3:**
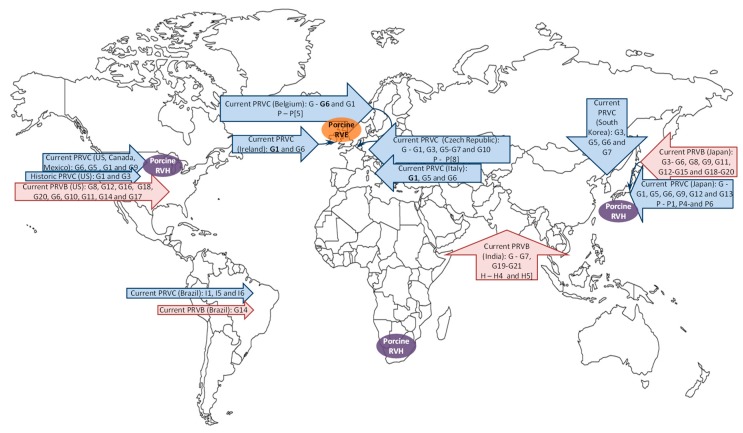
Global genotype distribution of porcine RVB (pink figure arrows) and RVC (blue figure arrows) strains and porcine RVE (bolded, orange circle)/RVH (bolded, purple circles) occurrence in different countries reported in historic (1976–2011) and current (after 2000) studies. Porcine RVCs are also detected in Germany and China, and porcine RVB is confirmed in Germany and Czech Republic, but no porcine RVC/RVB genotyping data is available for these countries.

An earlier study by Kim et al. (1999) identified porcine RVC strains associated with diarrheal outbreaks in feeder pigs in the US [93]. Although the porcine RVC prevalence was not evaluated in this study, phylogenetic analysis demonstrated that the identified strains were more closely related to Cowden (G1) and more distant to HF (G3) strains. Recently, Amimo et al. reported a higher overall prevalence of porcine RVC strains compared to that of porcine RVA strains (19.5% versus 9.4%) in diarrheic and non-diarrheic piglets collected from several farms in the US (OH, NC and MI) in 2004–2012 [119]. In this study, the frequency of porcine RVC identification in the samples from diarrheic was higher than that in non-diarrheic piglets. The porcine RVC strains were confirmed as G3 and G6 in this study (Figure 3). Further, Marthaler analyzed 7520 porcine fecal samples (collected in 2009–2011 in the US and Canada) and identified RVC in 46% of the samples tested [94]. The porcine RVC prevalence was 16% in very young pigs (<3 days old), 21% in young pigs (4–20 days old), 42% in post-weaning pigs (21–55 days old), 13% in older pigs (455 days old), and 8% in pigs of unknown age. However, single porcine RVC infection prevalence was highest in very young (<3 days), and young pigs (4–22 days) in 78% and 65% of the RVC positive samples, respectively, whereas this percentage was much lower (6%–39%) in the older age groups. The most common VP7 genotype detected in this study was G6 (70%), followed by G5 (17%), G1 (12%), and G9 (1%); however, unlike in the study conducted by Amimo et al., no G3 strains were identified. These data suggest that despite the limited genotyping information available for porcine RVC strains, there was a possible shift in their prevalence from G1 and G3 genotypes associated with the prototype Cowden and HF strains to G6 and G5 genotypes.

The current knowledge of the genetic diversity of porcine RVB strains is mostly from two studies: Kuga et al. (2009) and Marthaler et al. (2012) from Japan and the US, respectively. They classified the existing porcine RVB strains into 20 G genotypes [110,111] (Figure 3). Due to the limited information on porcine RVB epidemiology, it is hard to provide an accurate statistics on the temporal fluctuations in porcine RVB prevalence and porcine RVB genotype distribution in the North and South Americas. However, the new findings reported by Marthaler suggest an increased porcine RVB prevalence (46.8%) in the US that was previously observed by others elsewhere and in the US [67,104,110,112], and demonstrate that remarkably diverse porcine RVB genotypes (10 G genotypes: G6, G8, G10, G11, G12, G14, G16, G17, G18 and G20 associated with various I genotypes) are currently circulating in the US, with G8, G12, G16, G18 and G20 genotypes being most prevalent [111]. 

### 3.2. South America

As reported for the US, G5, G4 and G9 genotypes of porcine RVA were most prevalent in Brazil and Argentina, with G5P[7] being the single most prevalent combination [68,72,82]. Similar to findings by Marthaler and Amimo, recent findings by Molinari (on samples collected from a single diarrheic outbreak in Brazil in a G5P[7] vaccinated herd in 2012) demonstrated that porcine RVC (78%) was the most prevalent group found in single (34%) and mixed (44%) infections, followed by porcine RVA (46%), RVB (32%), and RVH (18%) [112]. The porcine RVA genotypes detected were G5P[13] and G9P[23], that differed from the G5P[7] found in the vaccine. Another recent study from Brazil (2011–2012) demonstrated co-circulation of G3, G5, G9, and P[6], P[13]/P[22]-like, and P[23] genotypes [120], but with no indication of the historic G5P[7] genotype combination. These findings may indicate that application of the G5P[7] based porcine RVA vaccines in North and South America might have contributed to the previously reported increased prevalence of the G5P[7] strains, while subsequently developed herd immunity and selective pressure against the G5P[7] strains, resulted in their recent decline (or disappearance) and emergence of the G9 or reassortant variants. Similar to the findings by Marthaler [94], Molinari reported an increased prevalence of porcine RVC strains in Brazil in diarrheic piglets in a herd vaccinated with porcine RVA G5P[7] vaccine [112]. The VP6 gene sequence analysis demonstrated that the RVC strain possessed an I1 genotype like Cowden; however, G and P types were not determined. Another study from Brazil, confirmed the presence of three I genotypes (I1, I5, and I6) in the samples from diarrheic piglets (2004–2010) suggesting that diverse porcine RVC strains circulate in different Brazilian states [98]. Additionally, Molinari et al. reported porcine RVB genotype G14 in diarrheic pigs in Brazil, as also reported by Kuga and Marthaler [110,111,112].

## 4. Global Porcine RV Distribution and Genotype Prevalence: Africa, Europe, Asia and Australia

### 4.1. Africa 

The presence of group A, B, C and H porcine RVs has been confirmed in several African countries [65,117,121,122,123]. The prevalence of porcine RVA in Kenya and Uganda reported in the recent study by Amimo et al. of 26.2% [65] was higher compared to the prevalence rates of 6.5%–25.7% reported for samples collected in 2004–2011 in the USA [65], several European countries [75,84,85], Thailand [89], and India [124], but however, lower (32.7%–38.3%) to those observed in Vietnam [74], Brazil [80] and Korea [76]. It was lower than that reported for samples collected in the US in 2009–2011 [69], in asymptomatic pigs in Italy (71.5%) [70] or previously reported for South Africa (84.6%) [123]. This study provides the first evidence that porcine RVA infections are widespread and likely endemic in East African pig herds. The 18 characterized African porcine RVA strains were classified into three different P-types including P[6], P[8] and P[13] that were associated with G5 and G23 G-types [65] (Figure 2). An increased prevalence of porcine RVA strains in diarrheic and asymptomatic suckling and weaned piglets of 41.8% was also reported in Tanzania (2014), but the identified porcine RVA positive samples were not genotyped [125]. Interestingly, although previous attempts to characterize porcine RVA strains from piglets in Nigeria by classical serotyping methods demonstrated the presence of G4 and G5 types, substantial numbers of the strains from that study was non-typeable [122]. These findings indicate that phylogenetically distinct porcine RVA genotypes/strains may circulate in African countries together with the historically common (G4 and G5) genotypes and warrants further epidemiological investigation.

Apart from some data on porcine RVB and RVC prevalence in Africa reported by Geyer et al. nearly three decades ago [123], the absence of surveillance programs and adequate diagnostic facilities have resulted in a lack of data on porcine RVB and RVC prevalence and genetic composition [65]; however, recently Amimo and colleagues demonstrated 8.3% (37/446) prevalence of porcine RVC in swine populations in Kenya (8.8%) and Uganda (7.7%) (Amimo et al., 2014, unpublished data).

A recent discovery and characterization of a porcine RVH strain from diarrheic piglets in South Africa confirmed that it was closely related to Japanese, Brazilian and the US porcine RVH, but not human RVH or bat (RVH-like) strains [117,121].

### 4.2. Europe

Diarrhea associated with RVA, RVB and RVC infections in pigs is an important cause of increased mortality, growth impairment, and economic losses in Europe [73,85,126,127]. porcine RVA strains of G2, G3, G4, G5, G9 and G11 and P[6], P[7], P[13], P[23] and P[27] genotypes were isolated from feces of diarrheic and non-diarrheic Belgian piglets in 2012 [128] (Figure 2). A wide range of G/P genotype combinations including; G3P[6], G4P[6], G5P[6], G4P[7], G5P[7], G9P[7], G9P[13] and G9P[23] was commonly detected in stool samples of diarrheic and non-diarrheic pigs in Belgium. Additionally, uncommon genotypes/genotype combinations were reported; G2P[27], G11P[27] and G4P[11]. During a large surveillance study in Italy (2003–2004), a total of 751 fecal samples were collected from nursing and weaned pigs involved in outbreaks of diarrhea [70]. Porcine RVA prevalence of 16.1% was identified by electron microscopy or by a commercial immunoenzyme assay. Upon either PCR genotyping or sequencing, the porcine RVA strains displayed a broad spectrum of VP7 and VP4 types, including G2-like, G3, G4, G5, G6, G9, P[6], P[7], P[13], P[23], and P[26] [70,129,130]. However, an earlier study by Martella et al. (2001) demonstrated that porcine stool samples collected in Northern Italy during a massive diarrheal outbreak in 1983–1984 contained porcine RVA strains of G6P[5] genotype combination [127]. Furthermore, Midgley et al. (2012) analyzed a total of 1101 fecal samples from pigs collected from 134 swine farms in four European countries (Denmark, Hungary, Slovenia and Spain) in 2003–2007 [85]. The results demonstrated that porcine RVA prevalence in Danish swine was only 10% although all samples were collected from diarrheic animals. In contrast, in Slovenia where the majority of swine were asymptomatic, the porcine RVA detection rate (20%) was significantly higher than that in swine with diarrhea in Denmark. This is consistent with the results by Amimo et al. [67] showing that unlike porcine RVC [119], there was no strong association between diarrhea and porcine RVA prevalence in nursing and suckling piglets in the US. However, in Spain, porcine RVA infections were significantly more frequent in animals with diarrhea (27%) than in asymptomatic animals (7%) [75]. Among these porcine RVA positive samples, ten different G types, G1–6 and G9–12, and nine different P types, P[6], P[7], P[8], P[9], P[10], P[13], P[23], P[27], P[32], were detected. No single G type was found to be dominant across the participating countries. In Slovenia G3, G4, and G5 were all common genotypes detected in 19%–30% of the samples. In Denmark, G4 was the most common genotype (44%). G9 was only detected in Spain, where it was the most prevalent genotype (33%). Among the various P types, only P[6] was detected in all four countries, which was the most common type in both Slovenia (41%) and in Denmark (56%). Otto et al. (2015) reported a porcine RVA prevalence of 51.2%, but no genotyping data was available from this study [126]. Finally, of the three positive porcine RVA samples identified in the Netherlands in 1999–2001, two were determined to possess G4P[6] and one G3P[7] genotype constellations [131]. Collins et al. tested 292 fecal samples collected from 4–5- to 8–9-week-old asymptomatic pigs in Ireland (2005–2007) and showed that 6.5% samples were positive for porcine RVA [84]. By sequence analysis of the VP7 and VP4 (VP8*) genes, the Irish porcine RVA strains were identified as G2, G4, G5, G9 and G11 and P[6], P[7], P[13], P[13]/[22], P[26] and P[32] genotypes, respectively [84]. The G5 and G11 strains were closely related to other human and porcine G11 strains, while the G2 and G9 strains resembled porcine G2 viruses detected recently in Europe and southern Asia. However, the G4 strains were only distantly related to other G4 human and animal strains, constituting a separate G4 VP7 lineage. Winiarczyk et al. (2002) identified G3, G4 and G5 types in combination with P6 and P7 types circulating in Poland [118]. Thus, in most European countries no dominant porcine RVA genotype/genotype constellations or temporal fluctuations in their prevalence was identified; however, the findings by Martella from different years suggest some epidemiological changes over time in Italy: disappearance of G6P[5] genotype constellation in more recent compared to historic studies [70,127]. A study conducted in England between 2010 and 2012 on samples from diarrheic pigs also revealed the presence of a wide range of porcine RVA genotypes: six G types: G2, G3, G4, G5, G9 and G11 and six P types: P[6], P[7], P[8], P[13], P[23], and P[32] [132]. G4 and G5 were the most common VP7 genotypes, accounting for 25% (16/64) and 36% (23/64) of the strains, respectively, while P[6] (33%, 21/64) and P[32] (27%, 17/64) were the most common VP4 genotypes, respectively. Overall, the most common genotype combinations were G4P[6] and G5P[7], similar to those detected in the historic US samples emphasizing the current unique epidemiology of porcine RVA in England compared to other European countries. 

Porcine RVC strains have been detected in feces of asymptomatically infected 4–5 week old Irish pigs (in 2005–2007) and of diarrheic piglets from the Czech Republic at low rates of 4.4% (of 292 samples) and 4.6% (of 329 samples) [104,133]. In comparison 29% and 31% of diarrheic piglets in Belgium (2014) and Germany (1999–2011), respectively, were porcine RVC positive in recent studies [73,126]. All Belgian porcine RVC strains characterized in the study belonged to genotype G6, except for one strain possessing the G1 genotype, while the VP4 genes were genetically heterogeneous, but were classified in the genotype P5 [73] (Figure 3). The majority of the Irish porcine RVC strains were identified as G1 genotype, while only two strains belonged to the genotype G6 [104] and the German porcine RVC strains were not typed [126]. A higher genetic heterogeneity was reported among Czech porcine RVC strains that were grouped into six G genotypes (G1, G3, G5–G7, and a newly described G10 genotype) based on an 85% nucleotide identity cutoff value [103]. Analysis of the VP4 gene revealed low nucleotide sequence identities between two Czech strains and other porcine (72.2%–75.3%), bovine (74.1%–74.6%), and human (69.1%–69.3%) RVCs and was tentatively classified as a novel RVC VP4 genotype, P8 [103]. Martella et al. (2007) characterized 20 porcine RVC strains collected from distinct diarrheal outbreaks in 2003–2005 in Northern and Central Italy [97]. They belonged to G1, G5 and G6 genotypes, similar to those identified in Ireland.

A very low prevalence rate of porcine RVB was reported in Germany in samples collected between 1999 and 2013, with no genotyping data available [126]. Additionally, Smitalova et al. (2009) reported that porcine RVB was detected in 0.6% of samples from diarrheic pigs in Czech Republic [133]; but they were not genotyped. Apart from the above information, no data for porcine RVB prevalence, pathogenic potential and genetic characteristics are available for Europe. Additionally, no reports of porcine RVH are available and only one historic study confirmed circulation of porcine RVE in England [25], requiring further evaluation and verification that pigs in fact serve as natural reservoir for porcine RVE strains.

### 4.3. Asia 

Numerous prevalence studies conducted in Asian countries demonstrated the presence of uncommon RVA genotypes in humans suspected to originate from animal sources [134,135,136,137] and reassortants of human-animal origin [83,138] including the G9 strains emerging globally or regionally in pigs and humans and the need of careful monitoring of animal RVs. Teodoroff et al. (2005) reported that genotype G9 of porcine RVA was dominant in a survey among porcine RVA strains associated with outbreaks of diarrhea in young pigs in Japan between 2000 and 2002 [139] (Figure 2). Similarly, Miyazaki et al. (2011) demonstrated that G9P[23], G9P[13]/[22], G9P[23], G3P[7], G9P[23], G5P[13]/[22], and P[7] combined with an untypeable G genotype caused four different diarrheal outbreaks in Japan in 2009–2010 that affected almost all suckling pigs born to 20% to 30% of lactating sows [90]. Further, this study provided evidence that the untypeable G genotype was a novel porcine RVA G26 genotype [90], which was confirmed by the Rotavirus Classification Working Group. A large-scale surveillance study of smallholder pig farms in the Mekong Delta, Vietnam, was conducted in 2012 and demonstrated an overall animal-level and farm-level porcine RVA prevalence of 32.7% (239/730) and 74% (77/104), respectively; however, no significant association with clinical disease was observed [74]. The study also identified six different G types and four P types in various combinations (G2, G3, G4, G5, G9, G11 and P[6], P[13], P[23], and P[34]) [74]. Additionally, one G26 strain was detected. A novel genotype P[27] in combination with G2 was identified in Thailand in samples collected in 2000–2001 [140]. Saikruang et al. (2013) reported an overall prevalence of porcine RVA of 19.8% (of 207 samples) in diarrheic samples of piglets in Thailand (2009–2010) and identified a wider variety of G-P combinations [78]. In this study, G4P[6] was identified as the most prevalent genotype (39.0%), followed by G4P[23] (12.2%), G3P[23] (7.3%), G4P[19] (7.3%), G3P[6] (4.9%), G3P[13] (4.9%), G3P[19] (4.9%), G9P[13] (4.9%), G9P[19] (4.9%), G5P[6] and G5P[13] each of 2.4%. Furthermore, G5 and G9 in combinations with P-nontypeable strains were also found as 2.4% (*n* = 1) of the collection. Among the diverse porcine RVA strains, novel genotype combinations of G4P[19] and G9P[19] were detected for the first time. Further corroborating the emergence and widespread prevalence of non-classic G and P genotypes of porcine RVA in Asia, 92.9% of porcine RVA containing stool samples collected from piglets with diarrhea in northern Thailand (2006–2008) belonged to the rare P[23] genotype combination with G9 or G3 genotypes [89]. The G9P[23] combination was reported to circulate in pigs in China as well [141]. Porcine RVA strains of the G9 genotype in combination with the P[7] and P[23] genotypes were isolated and identified as the third most important genotype in the diarrheic pigs in South Korea, after G5P[7] and G8P[7] [93]. A review by Malik and colleagues summarized the results of various surveillance studies (using ELISA-, PAGE- and PCR-based typing) suggesting the presence of G4, G6, G9, G12 and P[6], P[7], P[13] and P[19] genotypes in different regions in India [124]. Although there are no documented large-scale surveillance programs in China, the presence of porcine RVA G9P[7] in piglets with diarrhea was confirmed in Jiangsu Province, China [142], suggesting that various G9 combinations circulate in most if not all Asian countries. 

Despite somewhat scarce information on porcine RVC prevalence in Asian countries, there are several reports describing different porcine RVC genotypes circulating in Japan and South Korea [99,100,105]. The genotypes described in Japan include G1, G5, G6, G9, G12 and G13 G genotypes found in combination with P1, P4–P6 P genotypes, while G3, G5, G6 and G7 G genotypes were shown to circulate in South Korea [100,105] (Figure 3). There is also a report of porcine RVC circulation in China with a prevalence rate of 16.65% among diarrheic and asymptomatic piglets (2007–2008); however, no genotyping data is available [143].

Similar to porcine RVC data, very limited information on porcine RVB prevalence and dominant genotypes circulating in most Asian countries is available. A high prevalence of porcine RVB and porcine RVB specific antibodies in porcine fecal and serum samples, respectively, are reported in several studies in Japan [110,144]. Furthermore, at least G3–G6, G8, G9, G11, G12–G15 and G18–G20 genotypes with distinct sub-clusters within the genotypes were identified in porcine samples collected in Japan between 2000 and 2007 [71,110] (Figure 3). Additional evidence of remarkable porcine RVB diversity is highlighted in a report from India that demonstrates that at least G7, G19, G20 and tentative novel G21 genotypes (associated with H4 and H5 genotypes) circulate in the Northern and Western regions of India [145]. 

### 4.4. Australia 

Apart from several reports on circulation of porcine RVA G3, G4 and G5 ~3 decades ago [146,147,148], there is no epidemiological data for porcine RVs in this region (Figure 2). 

## 5. Zoonotic Potential of Porcine RV Strains

Historically, RVs were believed to be host-specific; however, recent and growing evidence challenges this postulation. Diverse animal reservoirs of zoonotic RVs are suggested to include at least porcine, bovine, ovine, pteropine, rodent, avian and insectivore species [17,85,149,150,151]. The widely documented zoonotic potential of RVA strains is best exemplified by globally emerging human RVs, such as G9 and G12, likely originating from porcine species by gene reassortment because similar G9 and G12 VP7 specificities are often observed in piglets [139,152,153,154]. Additionally, numerous reports have described interspecies transmission leading to sporadic cases of human disease with RVs from different animal species origin [72,155,156,157,158]. Table 1 summarizes common (G1–G4, P[6] and P[8]) and uncommon human RV G and P genotypes (suggestive of possible emergence via re-assortment) and G/P combinations (indicating possible direct transmission) that likely originated from swine. A total of 10 G genotypes (G1–5, G9–G12 and G26) and 7 P genotypes (P[4], P[6], P[8], P[13], P[14], P[19] and P[25]) of porcine origin have been identified in humans to date, with some genotypes including G10, G11, G12, G26, P[13], P[14], P[19] and P[25] displaying regional characteristics (found only in Asian or African countries), whereas the rest were found more commonly or emerging globally (Table 1). The recent discovery showing that different P-genotypes of RVA strains interact with distinct histo-blood group antigens (HBGA, ABOH, Lewis) and sialic acids via VP4 may provide insights into regional prevalence and increased zoonotic potential of some RVAs of swine origin [159,160,161,162]. While only a few animal RVs (of P[1], P[2], P[3], and P[7]) are sialidase sensitive, cellular attachment of human and the majority of animal RVs are sialic acid independent and use HBGAs as attachment factors or (co)receptors [161]. Further, RVs bearing different P-types recognize polymorphic HBGAs in a strain-specific manner, leading to variable host-specific susceptibility among different populations. Further, a stepwise-biosynthesis of HBGAs may represent one of the mechanisms regulating age-specific susceptibility to RV infection in early life [161]. Similar polymorphic HBGAs are also observed in many animals, including pigs (A and H antigens) [163]. The latter may provide an explanation why RVA strains of the P[6] genotype (that recognize H antigen) are commonly found in and transmitted between humans and pigs in different countries, while P[19] strains in humans of potential porcine origin appear to be restricted to India, Asian and African countries coinciding with distinct polymorphisms in Lewis antigens associated with Caucasian and other populations [164]. 

Unlike porcine RVA strains which are commonly demonstrated to possess zoonotic potential [17], there is currently little evidence in support of porcine RVC interspecies transmission. Identification of porcine RVC-derived genes in human and bovine RVC strains was reported in Brazil [199]. In addition to identification of bovine RVC strain WD534tc of likely porcine origin [203], whole genome analysis of porcine RVC strains from Japan has suggested a close phylogenetic relations between the human and some of these porcine RVC strains [100]. Additionally, a possible zoonotic role of animal RVCs has also been hypothesized based on increased seroprevalence rates to RVC in human populations [7] and the high prevalence of RVC infections in some geographic areas where they may cause <5% of gastroenteritis-associated hospitalizations in childhood [204]. However, it is important to note that the limited genetic variability of RVCs in humans contrasts with the high genetic diversity currently seen in pigs [97]. 

More recently, RVB strains were identified from sporadic cases of infantile diarrhea in Bangladesh as opposed to adult diarrhea cases associated with RVB in China and India. These recent strains differed genetically from the Chinese strain [53,55], suggesting that diverse RVB strains are circulating in humans. Limited evidence for the zoonotic potential of some porcine RVB strains was provided by Medici and colleagues demonstrated a high nucleotide identity between the NSP2 gene sequences of human and porcine strains [198]. 

Overall, these data indicate that frequent surveillance of porcine RVA and additional research on porcine RVB/RVC diversity in swine are needed to control their regional and global zoonotic spread. 

## 6. Passive and Active Immunity

**Figure 4:**
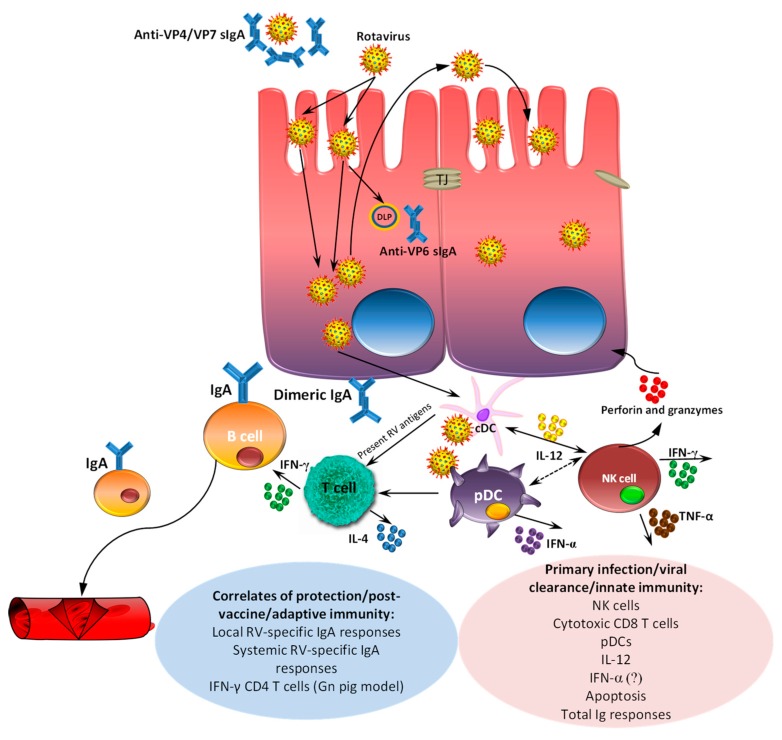
Immune responses to RV infection in pigs. Intestinal RV VP4/VP7 secretory immunoglobulin A (sIgA) neutralizing antibodies can prevent viral binding to enterocytes and penetration (early post-infection), while viral replication can be partially inhibited by anti-VP6 sIgA during transcytosis across enterocytes. In addition, a number of immune cells contribute to RV innate and adaptive immune responses: plasmacytoid dendritic cells (pDCs) produce antiviral (IFN-α) and pro-inflammatory (IL-12) cytokines which can inhibit RV replication or induce other immune cell subsets, including natural killer (NK) cells that produce granzymes, perforins and TNF-α and can lyse RV-infected cells. After antigen presentation by conventional dendritic cells (cDCs) to T cells, cytokine-secreting (IFN-γ in particular) RV-specific Th cells can also inhibit viral replication and activate IgA production by B cells. Additionally, RV-specific CD8 cytotoxic IFN-γ producing T cells contribute to the lysis of RV infected cells. RV induces apoptosis of intestinal epithelial (enterocytes) and immune cells; however, it is unclear whether this decreases (by eliminating infected cells) or promotes (via dissemination of the infectious particles) RV replication. Although high levels of systemic RV-neutralizing antibodies may coincide with improved protection against RV challenge, they are not correlated with protection in most studies. TJ: tight junctions. DLP: double-layered particles.

Immune responses and correlates of protection against RVs in humans and different animal species (mostly against RVA) are reviewed elsewhere [205,206]. Much of the knowledge of RV immune responses has been generated using a gnotobiotic (Gn) pig model and human RV infection/vaccines. In this review, we will briefly summarize passive and active immune responses in pigs induced by human RVA strains (Figure 4), since piglets can be infected with porcine and human RV strains, and develop clinical disease [206]. In terms of innate immunity, our recent studies have demonstrated that decreased severity of human RV clinical disease and infection was associated with enhanced function and frequencies of plasmacytoid dendritic cell (pDC) and natural killer (NK) cells evident systemically and locally and systemic IL-12 responses [207], similar to observations in humans and mice [208]. Although the role of interferon (IFN)-α in protection against homologous/heterologous RV infections is debated [209,210,211], earlier we demonstrated that an imbalanced IFN-α production coincided with increased human RV disease/infection severity [212]. Additionally, increased expression of toll-like receptor 3 (recognizes double-stranded RNA) was associated with improved protection against human RV infection and disease in Gn piglets, suggesting it could be an attractive target for therapeutic development [213]. Finally, reduced human RV replication in Gn piglets in our recent studies was associated with increased total Ig responses in systemic and local tissues [214].

The correlates of oral human RV vaccine induced protection against challenge with human RV (G1P[8]) were the presence and concentration of RV-specific IgA antibodies or antibody-secreting-cells (ASC) in serum or intestine, and frequencies of IFN-γ producing CD4+ T cells, but not the concentration of intestinal or systemic RV-neutralizing antibodies [215,216,217] or VP6-specific IgA antibodies [205,206,218] (Figure 4).

Priming orally with an attenuated human RV vaccine conferred protection in piglets that was augmented by a booster with VP 2/6 virus–like particles (VLPs) [218]. This protection was correlated with immune responses to VP4 and VP7 [206]. However, systemic and intestinal immune responses to human RV NSP4 alone did not correlate with protection of Gn piglets against human RV challenge [219]. While maternally derived circulating RV-specific antibodies mediated high levels of passive protection against human RV disease, active immune responses to replicating and non-replicating human RV vaccines were suppressed, as evident by reduced numbers of ASC in the intestine which decreased protection upon experimental challenge [220,221].

## 7. Porcine RVA Vaccines and Control Strategies: Potential Impact of Vaccines on Porcine RVA Genetic Diversity

Although following worldwide application of human RV vaccines, child mortality due to diarrhea declined, RV remains the most common cause of severe dehydrating diarrhea among children <5 years of age [222]. In livestock, vaccination strategies were focused on the induction of active or passive immunity, however, oral administration of attenuated RV vaccines to piglets and calves often lacked efficacy in the field [223]. The endemic porcine RV infections and the ubiquitous presence of porcine RV antibodies in swine revealed a need for strategies to boost lactogenic immunity in sows to provide passive antibodies to the neonate with colostrum and milk. The variable success of maternal RV vaccines in the field is influenced by vaccine dose, strain, inactivating agent, adjuvant, route of administration, and porcine RV exposure levels. The use of genetically engineered VLP vaccines to boost antibodies in mammary secretions showed promise because they are replication independent allowing circumvention of maternal antibody interference. However, although the immunogenicity of such VLP vaccines was high, the protective efficacy they induced was insufficient demonstrating the need for priming with live attenuated RV vaccines [224]. Nevertheless, field application of ProSystem porcine RV vaccine (which contained modified live porcine RVA strains of G4P[6] and G5P[7] genotype combinations) or G5P[7] (porcine RVA OSU) based vaccines could have resulted in the widespread circulation of porcine RVA of these genotypes for several decades and their more recent substitution by G9 and G11 genotypes or reassortant G4 and G5 variants discussed in detail in Section 3 of this review. Alternatively, they could generate herd immunity gradually decreasing the prevalence of the historic G4/G5 porcine RVA genotypes and allowing for the spread of novel emerging porcine RVAs.

## 8. Concluding Remarks

The remarkable diversity and genetic plasticity of porcine RVs indicate a need for further research on molecular characterization and spatio-temporal prevalence and fluctuations of endemic and emerging porcine RVs. The recent emergence of unusual G and P genotypes of porcine RVA strains worldwide, the discovery of novel porcine RV groups in different geographic regions, as well as the growing evidence of increased porcine RV prevalence and genetic diversity compared to that previously estimated suggest that porcine RV epidemiology is very complex and highly dynamic. These observations lead to at least two conclusions: (i) molecular diagnostic and characterization toolkits should be frequently updated and expanded to include novel porcine RV variants to ensure accurate epidemiological monitoring (especially for the countries where such information is lacking: African countries, Russia, Australia, etc.); (ii) a better understanding of the molecular pathogenesis and immunity to porcine RV is needed to optimize and update classical vaccine approaches to control porcine RV infections and spread. Although not highly efficacious in the field, attenuated replicating porcine RVA vaccines may be contributing directly to the genetic diversity of porcine RVs (via reassortment of vaccine strains with wild type strains and their subsequent spread) and the emergence of novel genetic variants that can evade herd immunity against the vaccine strains, as observed with human RVA vaccines, RotaTeq and Rotarix, that generate within vaccine (RotaTeq) and vaccine-wild type strain re-assortants capable of further spread in susceptible populations [225]. Alternative or additional approaches (to live attenuated vaccine use) may include wide-scale probiotic use or therapeutic applications that target the virus replication cycle to enhance innate or anamnestic immune responses, to decrease RV shedding and environmental contamination, and to alleviate porcine RV-mediated intestinal damage. Finally, although not previously well recognized, the zoonotic potential of various porcine RV genogroups/genotypes should be carefully and extensively evaluated by conducting simultaneous epidemiological studies of human and porcine RVs in the same geographic regions. Additional studies to understand the higher propensity of some genogroups/genotypes to generate re-assorted variants and cross interspecies barriers are needed, including the potential interactions of different porcine RV genotypes with HBGAs as shown for human RV strains [159,160,161,162].

## Figures and Tables

**Table 1:** Human RV genotypes of suspect or confirmed porcine origin via direct transmission or multiple re-assortment events.

Porcine RV Species	G and/or P Genotype	Geographic Region	Year Samples Collected	Epidemiological Status and Medical Relevance	Reference
**A**	**G1-G4, G9, G12 and P8**	Worldwide	2000s	Commonly seen in humans *	[152,153,165]
	**G3–G5, G9 and G11, as well as P[6]**	Denmark, France, Hungary, Italy, Slovenia	2003–2007	G3-G5—common in humans, G5—regional in humans, P6—rare in humans	[85]
	**G1 and G4**	Brazil	2007	Common	[166]
	**G1P[8]**	China; MD, USA	2004–2009	Common	[15,167,168]
	**G1, G1P[6]**	Japan	2001	Common	[169]
	**G1P[6]**	Japan	1997	Rare	[170]
	**G1P[6], G4P[6] and G12P[6]**	Democratic Republic of the Congo	2007–2010	Common	[171]
	**G1P[19]**	India	1992	Rare	[134]
	**G2**	Europe	1992	Uncommon	[130]
	**G3P[6], G4P[6] and G4P[8]**	China, Italy, Slovenia	2003–2013	Common	[172,173,174]
	**G3P[25]**	Taiwan	2009	Rare	[175]
	**G4P[6] strains, one G5P[6]**	Taiwan	2006–2012	Common	[176]
	**G4P[6]**	Hungary, China, Argentina, Madagascar	2006–2007 2008–2009	Sporadic identification in humans worldwide	[177,178,179,180,181]
	**G5P[6]**	Japan, Bulgaria	2011 2006	Rare	[182,183]
	**G5P[8]**	Brazil, Argentina, Paraguay, Cameroon, China, Thailand, and Vietnam	1986–2005	Common in Asian, African and South American countries	[184]
	**G9**	NE, USA; India	1980s, 1190s, 1997–2000	Uncommon, emerging worldwide	[153,185,186]
	**G9P[6]**	India	2007	Unusual	[187]
	**G9P[19]**	Thailand, India	2012–2013 1989–1990	Rare	[188,189]
	**G9P[19] and G9P[13]**	Taiwan	2014–2015	Rare	[190]
	**G9P[19] and G10P[14]**	Vietnam	2007–2008	Rare	[191]
	**G11P[4], G11P[6], G11P[8] G11P[25]**	Nepal, Bangladesh	2001–2004	Rare	[192]
	**G11P[25]**	India	2005–2009	Uncommon	[193,194]
	**G12P[6] and G12P[8]**	Kenya, Myanmar	2010, 2011	Common	[195,196]
	**G26P[19]**	Vietnam	2009–2010	Atypical in humans	[197]
**B**	**N/A**	Brazil	2000s	Regional significance	[198]
**C**	**N/A**	Japan, Brazil	1982–1986, 2000–2007	Regional significance	[100,199]

* G1P[8], G2P[4], G3P[8], G4P[8], and G9P[8]) were described in ~90% of samples from humans submitted to the EuroRotaNet database (that included data for 17 European countries: Belgium, Bulgaria, Denmark, Finland, France, Germany, Greece, Hungary, Italy, Lithuania, The Netherlands, Romania, Slovenia, Spain, Sweden, UK) between 2005 and 2009 from the 16 participating countries [200,201,202]. Letters of different colors represent different G-genotypes for easier distinction.
